# The community is just a small circle: citizen participation in the free maternal and child healthcare programme of Enugu State, Nigeria

**DOI:** 10.1080/16549716.2017.1421002

**Published:** 2018-01-17

**Authors:** Daniel C. Ogbuabor, Obinna E. Onwujekwe

**Affiliations:** ^a^ Department of Health Administration and Management, University of Nigeria Enugu Campus, Enugu, Nigeria; ^b^ Department of Health Systems and Policy, Sustainable Impact Resource Agency, Enugu, Nigeria; ^c^ Health Policy Research Group, Department of Pharmacology and Therapeutics, College of Medicine, University of Nigeria Enugu Campus, Enugu, Nigeria

**Keywords:** social accountability, free healthcare, policy implementation, Nigeria

## Abstract

**Background**: There is a gap in knowledge about how citizen participation impacts governance of free healthcare policies for universal health coverage in low- and middle-income countries.

**Objective**: This study provides evidence about how social accountability initiatives influenced revenue generation, pooling and fund management, purchasing and capacity of health facilities implementing the free maternal and child healthcare programme (FMCHP) in Enugu State, Nigeria.

**Methods**: The study adopted a descriptive, qualitative case-study design to explore how social accountability influenced implementation of the FMCHP at the state level and in two health districts (Isi-Uzo and Enugu Metropolis) in Enugu State. Data were collected from policymakers (*n* = 16), providers (*n* = 16) and health facility committee leaders (*n* = 12) through in-depth interviews. We also conducted focus-group discussions (*n* = 4) with 42 service users and document review. Data were analysed using thematic analysis.

**Results**: It was found that health facility committees (HFCs) have not been involved in the generation of funds, fund management and tracking of spending in FMCHP. The HFCs did not also seem to have increased transparency of benefits and payment of providers. The HFCs emerged as the dominant social accountability initiative in FMCHP but lacked power in the governance of free health services. The HFCs were constrained by weak legal framework, ineffectual FMCHP committees at the state and district levels, restricted financial information disclosure, distrustful relationships with policymakers and providers, weak patient complaint system and low use of service charter.

**Conclusion**: The HFCs have not played a significant role in health financing and service provision in FMCHP. The gaps in HFCs’ participation in health financing functions and service delivery need to be considered in the design and implementation of free maternal and child healthcare policies that aim to achieve universal health coverage.

## Background

Citizen participation is an important strategy for addressing persistent weaknesses in public sector performance and service delivery, and for making health systems more people-centred in low and middle-income countries [1,2]. An advanced form of citizen participation termed social accountability [3] comprises strategies, processes or interventions used by citizens to ensure that politicians, policymakers and service providers respond to citizens and account for their actions and decisions [4,5]. Social accountability strives to improve institutional performance by bolstering both citizen participation in policies and service delivery, and the public responsiveness of state and organisations [6]. Social accountability would become effective when citizens collectively recognise that governments have obligations to provide services, and that citizens are entitled to receive services [1,7].

Free healthcare policies are crucially important to achieving universal health coverage (UHC) in resource-constrained countries [8]. In the current era of sustainable development goals, with a huge focus on UHC, citizen participation in implementing health financing policies may promote service use relative to the need for care, responsiveness of providers and financial protection [9]. Social accountability initiatives, such as patient complaints procedures, health facility committees (HFCs), provider report cards and patients’ rights charters [10–13], may inspire users to participate in management of health facilities, complement government-led supervision and regulation, and improve the quality of healthcare decisions by government [4,6,14]. These accountability initiatives may improve the capacity of policymakers and providers to make changes to services based on citizens’ expectations [12,15], promote transparency in service entitlement and enhance health system outcomes [2,16].

Evidence of influence of social accountability in free healthcare policies in low- and middle-income countries is mixed and context-sensitive. Citizen participation in designing benefit, governing bodies, public hearing, satisfaction survey and call centre improved priority setting and contributed to increased revenue generation and termination of co-payment in Thailand’s universal coverage scheme (UCS) [17,18]. In contrast, participation of community representatives and users in allocating resources in Mexico’s Seguro Popular at the national, state and municipal levels was limited [19].

The users of free healthcare services in Thailand and Mexico had good knowledge of benefits and patient complaint service [17,20], which contrasts with evidence in India and Nigeria [21–23]. However, an information gap, collective action problems and lack of highly motivated citizens constrained the responsiveness of providers in Mexico’s Seguro Popular [24]. The use of complaint services was limited by an absence of complaint procedures, lack of knowledge of grievance redress and fear of reprisal or of being misunderstood in the Democratic Republic of the Congo and Tanzania [5,25]. In Kenya, the use of service charters was constrained by illegibility and language issues, lack of expenditure records, lack of time to read and understand them and socio-cultural limitations [26].

Gaps exist in the literature on the role of HFCs in governing free health services. Health facilities with strong, supportive and skilled health committees enhanced implementation of free healthcare policy in Nepal [27]. The use of HFCs in financial management of free healthcare policies in health centres in Burkina Faso and Kenya increased transparency in the fund management [28,29]. Although, Onwujekwe et al. [23] found a lack of community involvement in a free maternal and child healthcare policy in Nigeria, the study did not provide evidence of how the HFCs influenced the health financing functions (revenue generation, management of FMCHP fund and purchasing) and responsiveness of service providers.

In December 2007, Enugu State, Nigeria, introduced the free maternal and child healthcare programme (FMCHP) following adoption of user-fee removal for pregnant women and children as strategy for reversing the high maternal and child mortality rates by Nigerian government in 2006 [30]. The programme aims to reduce the risk of financial impoverishment for all pregnant women and children under 5 years and improve their use of public health facilities. User fees for maternal health services contribute to high patronage of faith clinics and traditional medical practitioners increasing the likelihood of avoidable deaths from pregnancy and childbirth [31]. Even when user fees are not impoverishing, financial constraints still significantly limited access to maternal health services in rural Nigeria [32,33]. Similarly, children under 5 years and from poor households are more likely to die than children from rich households in Nigeria [34]. However, despite committing to contributing 50% of annual FMCHP estimate each, the level of funding has stagnated since 2008, and the contributions from State and Local governments to FMCHP fund have been unpredictable [35,36]. Still, entitlement to free services has been linked to evidence of tax payment since 2011. Users who are unable to present evidence of tax payment paid providers directly for services, which seemed to create an incentive for providers to under-provide free services.

The FMCHP policy envisaged two social accountability strategies, namely HFCs and complaint systems, but was silent about the service charter. The HFCs were designed to monitor the delivery of free services, identify eligible users, provide platforms for consultations with citizens, raise awareness about free services, mobilise communities to use public health facilities, manage facility resources and facilitate implementation of the complaint systems including complaint boxes, hotlines, patient exit and vignette surveys in health facilities. Besides, the operational guidelines for HFCs provides for the HFCs to be involved in joint problem analysis and planning with other stakeholders at the facility and policy levels [37].

The HFCs consist of facility staff and community representatives, a third of whom must be women. The HFC members are selected by their communities for a renewable term of 3 years and must also include youths, people living with HIV/AIDS, people with disability and community-based organisations. The meetings are held at least monthly, but HFC members are not paid transport costs, sitting allowances or salaries. Instead, communities are encouraged to reward them in the ways they considered appropriate. The HFCs received training and mentorship from civil society organisations (CSOs), health development partners and Ministry of Health to execute their roles and form HFC Alliances at the local government and state levels. The HFC Alliances represent citizens in FMCHP committees at different levels of the health system.

Nonetheless, knowledge about the functioning of social accountability interventions in Enugu’s FMCHP is limited. It is also not clear how the HFCs participate in health financing functions and delivery of free services. Anecdotal evidence indicates that the FMCHP committees for fund management and purchasing, which should include HFCs’ representatives, are moribund [35]. As a result, the Ministry of Health pools and manages the FMCHP fund and pays providers for free services. Understanding how and why HFCs’ participation influences FMCHP implementation would provide decision-makers useful insights into the gaps that must be filled to ensure that the free care policy contributes to UHC.

This paper contributes to the literature on how social accountability impacts implementation of free healthcare policies in low- and middle-income countries. It provides evidence of how the HFCs enabled or constrained revenue generation, pooling and fund management, purchasing and capacity of health facilities implementing free maternal and child health services in Enugu State, southeast Nigeria.

## Methods

### Conceptual framework

**Figure 1.**
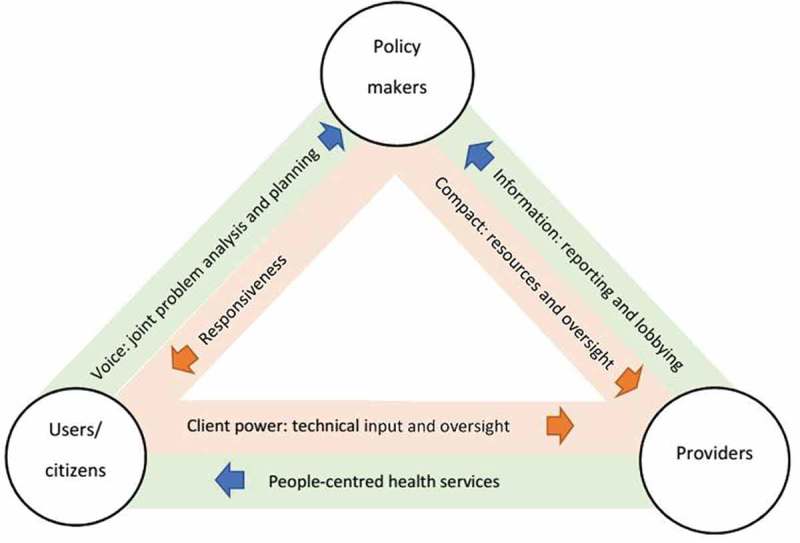
Conceptual framework of the study.

The study adopted the Bossert and Brinkerhoff health governance framework [38,39]. The framework focuses on diverse health systems actors, distribution of roles and responsibility among them, and their ability and willingness to fulfil these roles and responsibilities. The framework uses the principal-agent theory to explain accountability relationships involving three categories of health system actors: decision makers, providers and users/citizens [39]. Decision makers are policymakers in the public service. Service providers include health facilities and health workers. Users/citizens include service users and HFCs. These actors are in three accountability relationships: users/citizens-policymakers; users/citizens-providers; and policymakers-providers (Figure 1). We focused on the agency relationships involving users/citizens because they explain accountability relationships of the HFCs with policymakers and providers.

Social accountability is analysed in relation to HFCs because FMCHP design adopted HFCs as the main social accountability initiative. Additionally, HFCs use complaint boxes and service charters. Thus, the agency relationships of HFCs with policymakers and providers were analysed using the five modes of functioning of HFCs – village square, community connector, bothering government, back-up government and general overseer – which had been identified in two previous studies in Nigeria [40,41]. Village square implies that the HFCs use meetings as a vehicle for addressing issues and resolving challenges facing health facilities. As community connectors, the HFCs reach out within their communities and serve as a platform for citizens to share their views about the functioning of health facilities. The HFCs functioning as ‘bothering government’ bother policymakers to address problems in their facilities or programmes. The HFCs mobilise resources and fill service delivery gaps when they function as ‘back-up government’. General overseer means that the HFCs oversee day-to-day running of health facilities, participate in decision-making and monitor implementation of FMCHP.

### Study setting

**Figure 2.**
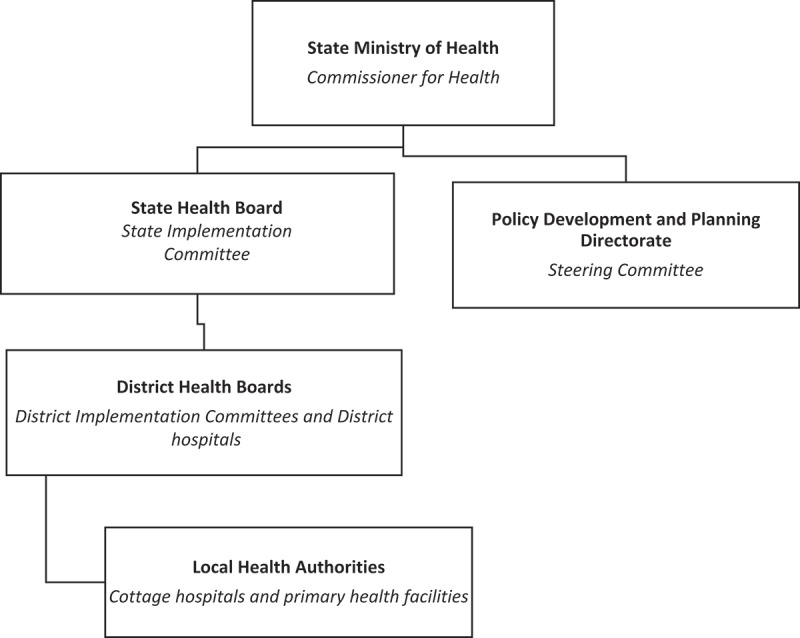
Enugu State District Health System.

The study was undertaken at the State Ministry of Health and in two selected districts (A = Isi-Uzo and B = Enugu Metropolis) in Enugu State, southeast Nigeria. Enugu State operates a district health system in which the 17 LGAs were delineated into seven districts and primary and secondary healthcare integrated within districts, to serve populations ranging from 160,000 to 600,000 persons [42]. The Ministry of Health is restructured into two arms: (1) the Policy Development and Planning Directorate, which houses the FMCHP Steering Committee, is responsible for leadership and governance; and (2) the State Health Board, which houses the State Implementation Committee, coordinates service delivery across the districts (Figure 2). Each district is governed by a district health board and has several local health authorities and network of health facilities providers including primary health centres and cottage and district hospitals. The contraceptive prevalence rate is 31.4%, total fertility rate is 4.8, access to skilled birth attendance is 38%, vaccination coverage is 47%, maternal mortality is 576 per 100,000 livebirths, and under-5 child mortality is 131 per 1000 livebirths in Enugu State [43].

### Research design

We adopted a qualitative, case-study design using document review, in-depth interviews (IDIs) and focus-group discussions (FGDs). Case-study design was used because the inquiry focused on ‘what, how and why questions’ [44,45].

### Study population and sampling strategy

The seven health districts in Enugu State were categorised into well-performing and less-performing districts based on provider payment data. We calculated the cumulative provider payment across the seven districts from financial records between 2009 and 2014 and found a range of 2% to 26% with a median of 14%. Using a cut-off point of 14%, three districts were adjudged to be well performing and four less performing. Provider payment was used to judge the success of FMCHP in districts, since state health information system does not disaggregate data by user-fee exemption. From each category, one district was selected by simple random sampling. The respondents from the state level were selected purposively from a list of members of the Steering Committee and State Implementation Committee. District-level policymakers, providers and HFC members were purposively selected based on their location, post and experience in FMCHP, and interviewed until data saturation was reached [46]. Participants, who had less than one-year involvement in FMCHP implementation at state level or selected districts were excluded from interview.

Maximum variation sampling was used to recruit 42 women who participated in four focus groups [46]. The participants were women of child bearing age who were 15–49 years, had at least one under-5-year child and were willing to participate in the study. In District A, two communities were selected randomly from a sampling frame of 20 autonomous communities. Working with community women leaders (gatekeepers), the study was advertised during the ‘August’ meeting (women gathering) and participants conveniently selected. In District B, it was more practical to reach urban women in health facilities on immunisation days than through a community approach adopted in district A. One primary health centre and one hospital each were selected randomly from sampling frames of primary health centres and hospitals. The participants were conveniently selected by advertising the research during immunisation day at the health facilities using service providers as gatekeepers.

### Data collection

Data were collected using document review, IDIs and FGDs between February and September 2015 during an assessment of governance of FMCHP in Enugu State, Nigeria. Information about social accountability initiatives was extracted from 14 policy documents. Interviews with 44 participants (16 policymakers, 16 providers and 12 HFC leaders) were conducted using a semi-structured in-depth interview guide. The guide explored the role played (or not played) by HFCs in revenue generation, pooling, purchasing and capacity of health facilities during implementation of FMCHP (see Appendix I). Interviews, lasting 60–90 minutes, were conducted in English and tape-recorded. Member checks were used to ensure that participants reviewed their statements for accuracy [47].

Four FGDs were held with a total of 42 women of childbearing age using a discussion guide (see Appendix II). Two focus groups were held in District A with 11 and 12 participants, while two focus groups in District B had eight and 11 participants respectively. The FGDs, which were audiotaped with the consent of participants, were held at venues chosen in consultation with participants and gatekeepers, moderated by one of the authors, and a research assistant served as note taker.

### Data analysis

Data were analysed using thematic analysis. Audiotapes of the interviews were transcribed verbatim, anonymised and imported into NVivo 11 software [48]. Codes were generated by deductive and inductive process, and defined in a codebook to minimise inter-coder differences [49]. Deductive codes were guided by the conceptual framework and included accountability relationships and modes of functioning of HFCs. Inductive codes, based on a close reading of the transcripts, highlighted the roles (not) played by HFCs in holding policymakers and providers accountable, and the context of these accountability relationships. Two persons coded the transcripts with much agreement. To ensure trustworthiness of findings, the research team was trained in qualitative research approaches, data-collection tools were pre-tested in a different district, findings were triangulated by methods, and inter-coder differences were resolved by unanimity. Member checks and stakeholder validation meetings were also used to verify the accuracy and completeness of findings [50].

### Ethical consideration

Ethical approval was obtained from the Health Research Ethics Committee of University of Nigeria Teaching Hospital Enugu, Nigeria. The participants gave written informed consent for participation and digital recording of interviews.

## Results

The socio-demographic characteristics of participants are summarised in Table 1.

**Table 1.** Socio-demographic profile of the participants.

Participants	Post/location	Total no.	Male	Female
Policymaker	State Ministry of Health (Policy Development and Planning Directorate)	5	3	2
	State Health Board	5	5	
	District level	6	5	1
Providers	Heads of health facilities	16	3	13
Citizens	Health facility committee leaders	12	11	1
Users	Women of childbearing age	42		42

## User/citizen–policymaker relationship

### Bothering government

Bothering government emerged as the only theme in user/citizen-policymaker relationship with four sub-themes: revenue generation, pooling and fund management, purchasing and responsiveness of service providers (Table 2).

**Table 2.** Main roles, activities and context of health facility committees in FMCHP.

Accountability relationship	Themes	Sub-themes	Roles (not) played	Contextual factors
HFC–policymaker relationship	Bothering Government	Revenue generation	Lack of HFC participation in fund raising and budgeting for FMCHP	HFCs not represented in Steering Committee
			HFCs not advocating for state budget transfer	Dysfunctional Steering Committee
		Pooling	HFCs not involved in fund management decision	Weak enforcement of spending rules
			HFCs not advocating financial disclosure	Weak HFC Alliance
		Purchasing	HFCs not involved in formulating evidence of tax policy (ETP)	
			HFCs advocating delinking of service entitlement from ETP	Presence of development partners
		Responsiveness of service providers	Advocate to government to engage volunteer health workers	HFCs with educated members
				HFC-policymakers trust
HFC–provider relationship	Village Square	Responsiveness of service providers	HFC meetings include FMCHP on agenda	Wide variations in attendance, punctuality and regularity of meetings
			Feedback to users and citizens	Availability of rewards
HFC–provider relationship	Community Connectors	Responsiveness of service providers	Address demand-side barriers to FMCHP	Availability of phone numbers of HFC members
			Resolve complaints	HFCs network with women groups
			Increase awareness of free services	Insufficient public communication
				Unavailability of complaint box
				Low awareness of complaint procedure among users
				Preference for verbal complaints
HFC–provider relationship	Back-up government	Responsiveness of service providers	Community fills service delivery gaps in health facilities	Supportive town union/traditional rulers
				Community ownership of HFCs
HFC–provider relationship	General Overseer	Responsiveness of service providers	Monitor staff attendance	Trained and mentored HFCs by civil society organisations
				Presence of development partners
			Monitor drug-revolving fund	Willingness of providers to involve HFCs
			HFCs monitors use of free care funds in health facilities	Service charter exists but unavailable in health facilities
			Increase utilization of free care	Healthcare providers’ indifference/clients’ lack of trust in public health institutions

It was found that the HFCs played no role in revenue generation. Despite support from CSOs and development partners, the HFCs did not advocate increased funding for FMCHP or timely state budget transfer to the FMCHP fund. Policymakers explained that although FMCHP preceded the formation of HFCs, the HFC Alliance had so far not engaged with decision makers effectively. Providers, HFC leaders and users did not know the rules for contribution and were not aware of the role of HFCs in revenue generation for FMCHP. Some HFC leaders even thought, wrongly, that FMCHP was being funded by health development partners, as illustrated by the words of one HFC leader, who also demonstrated a lack of information on which level of government has been funding FMCHP: ‘It seems that some donors are funding free MCH program and not just the state government. I don’t think that local governments are playing the role expected of them in funding the free MCH program’ (HFC leader 12).

Citizens are not involved in decision-making about the FMCHP fund. The HFCs were not involved in managing FMCHP funds or in demanding FMCHP financial information disclosure to the public. ‘HFC are not represented on Steering Committee and so have no platform for involvement in fund management’ (Policymaker 9). The Steering Committee is a multi-stakeholder committee assigned pooling and fund management function. In practice, the Steering Committee was moribund. Providers, HFC leaders and users were not aware of the role of HFCs in pooling and managing FMCHP funds. As one HFC leader put it, ‘HFCs are not involved in making decisions about FMCHP fund’ (HFC leader 12). Indeed, HFC leaders and service users did not know the rules guiding pooling and FMCHP funds administration. HFC leaders said that consumers have had no explicit role in FMCHP fund management, but explained that the HFC Alliance was set up to track FMCHP expenditure management, and that the HFC Alliance has so far not been effective in doing so.

The HFCs have not been involved in identifying eligible users and implementing evidence of tax payment policy as eligibility criterion for benefiting from free services. One HFC leader stated: ‘Members of HFCs were not involved in deciding the policy on evidence of tax payment. If we were involved, we could have suggested many other ways of identifying beneficiaries’ (HFC leader 12). This was because, as one HFC leader said, ‘If you ask anybody to go and bring evidence of tax payment before accessing care, you will never see the person again’ (HFC leader 4). Indeed, some policymakers indicated that HFCs and CSOs have been championing delinking of evidence of tax payment from service entitlement. One policymaker said, ‘In most of the meetings I attended, we have a woman called “voice of the voiceless”. Each time we met, she will shout: remove this evidence of tax policy. If you don’t remove it, it’s no longer free’ (Policymaker 8). However, the role of HFCs in provider payment has been limited. HFC leaders and service users are not aware of who the purchaser is, the method of provider payment adopted and the rules for reimbursement. One HFC leader said: ‘Whatever expenditure claims the providers submit to government, they don’t tell us whether the government paid them or not’ (HFC leader 4).

Nonetheless, the HFCs bothered government officials to fill service delivery gaps in health facilities. The HFCs mainly engaged with district-level decision makers, directly or through the HFC Alliance. Key issues presented to policy makers were infrastructure, security and staffing needs to support effective maternal and child health service delivery. But success has been limited, partly because, as explained by a policymaker, ‘Unless for the enlightened ones, they may not have that confidence to go straight to the policymakers to lay a complaint or to make a request’ (Policymaker 12) However, in an example of successful lobbying, one healthcare provider said: ‘There was one midwife… who was not employed but we needed her services… and she has been coming there to serve the community. The facility health committee chairman presented the issue to the local government and they employed her.’ (Provider 7)

## User/citizen–provider relationship

The HFCs functioned in four modes, namely village square, community connectors, back-up government and general overseers, to influence the responsiveness of service providers (Table 2).

### Village square

The HFC meetings functioned as a forum for representatives to interact and discuss FMCHP issues and other challenges facing the health facility, and ‘they [challenges] are addressed at the meetings and the issues that are beyond them are reported to higher levels’ (Policymaker 3). However, there were wide variations in effectiveness of meetings. Policymakers, providers and HFC leaders, however, noted that the effectiveness of meetings was limited by poor attendance and irregular timing of meetings. One HFC leader said, ‘We used to have meetings every month, but for three months now, we have not had our meeting’ (HFC leader 5). Unmet expectations for rewards for participation in meetings by the HFC members, who had such expectations, demoralised committee members and limited this mode of HFC functioning. In the words of one policymaker, ‘Since they (HFCs) are not receiving anything from anybody, it really hampers their involvement’ (Policymaker 2). And the HFC members complained that providers ‘are not giving them anything for the work they are doing’ (HFC leader 2); while providers shifted the blame elsewhere: ‘they (HFCs) are not paid by the government’ (Provider 6).

### Community connectors

The HFCs provide platform for mobilising users of FMCHP, networking with community groups and resolving complaints of users in health facilities. In the words of one policymaker, ‘They have tried to mobilise their citizens. They have tried to educate them. They have tried to address certain things that have hindered them from accessing free maternal and child healthcare’ (Policymaker 4). Community members provide information to the HFCs, creating circles of consultation. One HFC leader described their engagement with women group: ‘We rubbed minds with the women who are the main target of this free maternal and child healthcare programme’ (HFC leader 4). Community members also complain to the HFCs: ‘if a patient comes into the facility and is not well attended to, they always call us because they have our numbers; most of them know us’ (HFC leader 11). However, some policymakers and providers argued that the HFCs have not raised enough awareness of FMCHP in communities: ‘They have not played the role of sensitizing their communities about need to assess free services… You can’t leave where you have free treatment provided by well-trained people and you go to traditional health attendant to deliver’ (Policymaker 6). In addition, only a few HFCs explained policy changes in FMCHP to citizens, especially the requirement to present evidence of tax payment as a condition for accessing free services.

Participants of all categories observed that most health facilities lacked complaint box, and where complaint boxes were available, use was minimal. One user observed: ‘They [health facilities] do not have [a] suggestion box. If they have, I have not seen one since I started using services here’ (User, FGD 3). One policymaker said, ‘We are supposed to have [an] opinion box in every health facility but in truth, we don’t’ (Policymaker 14); while another was more sceptical of their value, saying ‘Suggestion box[es], do they work? They don’t work!’ (Policymaker 11). Instead, users complained through trusted HFC members or providers. One provider observed: ‘What we get mostly are verbal complaints’ (Provider 14). And according to another provider, ‘The community is just a small circle; they know the HFC chairman. They can complain through him or through any of the members’ (Provider 2). Nevertheless, the HFC and community members highlighted that having a complaint box will further ease the process. For example, one HFC leader said ‘It is important for us to have one so that patients can make suggestions. During our meeting, we can analyse them and see people’s opinions’ (HFC leader 2).

### Back-up government

Few HFCs mobilised resources from communities and filled the gaps in government support to health facilities. The HFCs engage community youths to keep surroundings of health facilities clean, employ private security staff to guard health facilities and ensure communities construct placenta pits or donated alternative power supply. In the words of a policymaker, ‘HFCs see some of the problems FMCHP encounters before it gets to us; they resolve a lot of them’ (Policymaker 7). Notably, those HFCs functioning in the back-up government mode do have strong town union, supportive traditional rulers, the presence of development partners and a functioning complaint mechanism. One provider noted that the HFC is ‘headed by the president-general of [name] union and has three traditional rulers as members… It was a strong committee to the point that the committee donated a giant power generating set to our health facility’ (Provider 1).

### General overseers

There were two areas in which the HFCs have been involved in management of health facilities, namely drug revolving fund (DRF) and monitoring of staff attendance to work. HFCs endorse the purchase of drugs to replenish the DRF stock, take record of all drugs that are procured and are co-signatory to the DRF account. In Isi-Uzo, a provider said ‘The HFC approves drug stock replenishment, and each time we procured, the Committee took stock before we dispensed new drugs’ (Provider 5). In Enugu Metropolis, a provider said ‘the HFC members are involved in monitoring of DRF; they check our stock during our monthly meeting’ (Provider 7). And a HFC leader said, ‘If the officer-in-charge pays money generated into drug revolving fund, I do get alert[ed] as the health committee chairman’ (HFC leader 9). In other instances, the HFCs check movement of staff and reported any staff, who are absent from duty to the local government. Staff salaries are sometimes not even paid until the HFC chairman has ‘endorsed staff time book’ (HFC leader 4). Training for the HFC members to perform these functions, presence of development partners who encourage them and supportive health facility officers-in-charge were the enabling factors for the HFCs functioning in the general overseer mode. A lack of complaint boxes and service charters weakened the capacity of the HFCs to hold providers accountable.

## Discussion

This study revealed a lack of participation of the HFCs in revenue generation, which contradicts evidence from Thailand’s UCS, where users are represented in the governing body and insist that government use evidence of utilisation and unit costs to calculate capitation rates [17,18]. Three reasons could explain this lack of citizen participation in revenue generation. First, the HFCs have a low awareness of the level of funding of FMCHP. Second, when the HFCs were reformed and HFC alliance established, the Steering Committee had become moribund. Third, the HFCs could not engage the Ministry of Health to reconstitute the Steering Committee due to a weak legal framework for the HFCs’ participation in FMCHP. In a setting where many people are living in poverty and are likely to forgo care or face financial hardship, the HFCs’ engagement in revenue generation is likely to stimulate State and Local governments to prioritise FMCHP funding. Improving social accountability in revenue generation in FMCHP would entail that the HFCs participate in the FMCHP Steering Committee and use the platform to advocate for sufficient funding and timely release of promised funds by governments, since reliance on public funding for health services is needed to progress towards UHC [9].

This study indicated that the HFCs have not been involved in decision-making about FMCHP funds, which is similar to evidence from Mexico where citizen participation in allocating resources in Seguro Popular was limited [19]. In contrast, our finding differs from evidence from Thailand’s UCS where user representatives are involved in fund management decisions [17]. These differences could be explained by differing contexts, which shape how health system actors respond to social accountability initiatives. A lack of participation of the HFCs in fund management resulted from a moribund Steering Committee and unwillingness of Ministry of Health officials to involve the HFCs. Also, the HFCs seem to have limited resources to track spending from FMCHP funds due to restricted financial information disclosure and distrustful relationship with public officials. In a climate where adherence to public financial management rules is low, the participation of the HFCs in FMCHP fund management could ensure that funds are available for provider payment and are properly accounted for, reported upon and monitored. When there are sufficient funds in the FMCHP account, providers can be paid timely for services delivered.

This study also showed that the HFCs did not seem to have increased the transparency of benefits and obligations in the FMCHP. Our finding is consistent with evidence from India and Nigeria [21–23], but differs from evidence in Thailand and Mexico where knowledge of benefits is high among users [17,20]. The HFCs did not seem to create sufficient awareness of evidence of tax payment among users in our study. Even where knowledge of evidence of tax payment seemed good, the poor were not able to pay taxes. As a result, providers modified the free care policy by resuming user fees, informal payments, service delays and denials. The programme modification seemed worse in district A than in district B due to a higher level of informality and may have contributed to a lower provider payment in district A. Nevertheless, as evidence of tax payment was introduced to ensure that only true residents of the state benefited from the scheme, the HFCs could have identified eligible users using other strategies. For example, eligibility can be confirmed through certification letter by community leaders, public utility bills and ensuring that users register with their local providers, as is the case in Thailand’s UCS [17,51]. Thus, the HFCs’ engagement in designing and implementing benefits would help users to understand clearly both their entitlement and obligations.

This study further revealed that the HFCs did not play any role in provider payment process due to exclusion of HFC leaders in decision-making by policymakers and providers. In contrast, the HFCs are involved in a provider payment system for free healthcare in Burkina Faso and Kenya [28,29]. As purchasing is an important strategic lever in UHC, the HFCs could facilitate the registration of eligible users with their primary providers, endorse claims forms and participate in managing facility FMCHP accounts. These roles of the HFCs in service provision and provider payment not only needed to be explicitly written in the FMCHP policy, but also required, as Anwari et al. [52] observed, consistent implementation through stakeholder engagement, consensus orientation and information sharing with providers and communities, and training to avoid experiences in Kenya where the HFCs were inadequately trained to manage direct facility funding [29].

The functioning of social accountability initiatives in facilities implementing FMCHP was notably weak in this study. The HFCs in this study mostly functioned as general overseer, providing oversight of DRF and monitoring staff attendance. Yet, few HFCs functioned as ‘back-up government’ to fill service delivery gaps notably volunteer staffing, security and power supply. This study confirmed the contextual factors shaping functioning of HFCs identified in previous studies in Nigeria [40,41]. However, most health facilities lacked a complaint box, and where a complaint box was available, its use was minimal. Our finding is consistent with evidence from India, Nigeria, Democratic Republic of Congo, Tanzania and Mexico [21–23] but differs from evidence in Thailand where complaint services are effective [17]. Even though users in Mexico had good knowledge of complaint systems, information gaps and limited collective action constrained the effectiveness of the complaint system [20]. The reasons adduced for low use of a complaint box included low literacy level of clients, fear of reprisal and insufficient client communication by providers, similar to the findings from studies in Democratic Republic of Congo and Tanzania [5,25]. Unfortunately, absence of complaint box limited users of the FMCHP from providing feedback to providers of the care process, who instead relied on the community being ‘just a small circle’ to share their complaints with the HFC members with whom they are familiar. Other dissatisfied clients resigned themselves to fate or patronised private healthcare providers.

Availability and use of service charter in health facilities and awareness among users and HFC members are low. The findings of this study contrast the high awareness of a service charter among users but consistent with the poor adherence to service charter provisions by health workers in Kenya [26]. The Enugu service charter is supported by state health law, which sets out the rights and entitlements for all patients attending public health facilities. However, apart from initial training of providers, little has been done to implement the service charter. Consequently, providers are not creating awareness about service charters among users and HFC members, who would monitor adherence of healthcare providers to service charter provisions.

This study contributes to policy debate on the role of social accountability in enhancing governance of health financing functions for UHC schemes. Social desirability bias, common among interview participants, could have limited this study, but this was avoided by in-depth probing and providing participants considerable assurances of confidentiality and anonymity [53]. Although the use of different sampling strategies to recruit FGD participants could bias the study, the purpose of the study was not to compare different districts but to understand how citizen participation is seen and experienced among different people, in different settings and at different times, thus maximising the diversity of experiences relevant to the research question.

## Conclusion

This study highlights the gaps in participation of the HFCs in revenue generation, management of FMCHP funds, payment of providers, designing of benefits and delivery of free services. Implementation of social accountability strategies in FMCHP was constrained by weak capacity of the HFCs, ineffectual Steering and State Implementation Committees, and distrustful relationships between the HFCs and policymakers and providers. The findings show that even though communities are ‘just a small circle’ with great potential for citizen participation, ensuring social accountability will often require additional support by policymakers and service users in the form of active engagement of the HFC members in joint problem analysis and planning with other stakeholders in the FMCHP Steering and Implementation Committees and strengthening social accountability initiatives in health facilities. Such actions will improve transparency and accountability in free healthcare policy implementation and enhance the attainment of UHC goals of service use relative to the need for care, service quality and protection from financial hardship.

## Paper context

Little is known about how social accountability impacts implementation of free maternal and child health policy in Enugu State, Nigeria. This study highlights the gaps in participation of HFCs in enhancing effectiveness of health financing functions for free healthcare policy to contribute to UHC. Ensuring social accountability will require addressing the factors limiting the active engagement of HFCs in health financing functions and strengthening of facility-based social accountability initiatives.

## References

[1] Brinkerhoff DW, Wetterberg A. Gauging the effects of social accountability on services, governance, and citizen empowerment. Public Adm Rev. 2016;76:274–12.

[2] Lodenstein E, Mafuta E, Kpatchavi AC, et al Social accountability in primary health care in West and Central Africa: exploring the role of health facility committees. BMC Health Serv Res. 2017;17:403. 28610626 10.1186/s12913-017-2344-7 PMC5470232

[3] McNeil M, Malena C Demanding good governance: lessons from social accountability initiatives in Africa. Washington, DC: The International Bank for Reconstruction and Development and World Bank; 2010.

[4] Lodenstein E, Dieleman M, Gerretsen B, et al A realist synthesis of the effect of social accountability interventions on health service providers’ and policymakers’ responsiveness. Syst Rev. 2013;2:98. 24199936 10.1186/2046-4053-2-98 PMC4226265

[5] Mafuta EM, Dieleman MA, Hogema LM, et al Social accountability for maternal health services in Muanda and Bolenge Health Zones, Democratic Republic of Congo: a situation analysis. BMC Health Serv Res. 2015;15:514. 26593716 10.1186/s12913-015-1176-6 PMC4655451

[6] Fox JA Social accountability: what does the evidence really say? World Dev. 2015;72:346–361.

[7] Hulton L, Matthews Z, Martin‐Hilber A, et al Using evidence to drive action: A “revolution in accountability” to implement quality care for better maternal and newborn health in Africa. Int J Gynecol Obstetrics. 2014;127:96–101. 10.1016/j.ijgo.2014.07.002 25087502

[8] Yaogo M Free versus subsidised healthcare: options for fee exemptions, access to care for vulnerable groups and effects on the health system in Burkina Faso. Health Res Policy Syst. 2017;15:58. 28722559 10.1186/s12961-017-0210-z PMC5516835

[9] Kutzin J, Witter S, Jowett M, et al 2017 Developing a national health financing strategy: a reference guide. Geneva: World Health Organisation (Health Financing Guidance No 3) Lincence: CC BY-NC-SA 3.0 IGO Available from: http://apps.who.int/iris/bitstream/10665/254757/1/9789241512107-eng.pdf

[10] Cleary SM, Molyneux S, Gilson L Resources, attitudes and culture: an understanding of the factors that influence the functioning of accountability mechanisms in primary health care settings. BMC Health Serv Res. 2013;13:320. 23953492 10.1186/1472-6963-13-320 PMC3844434

[11] Edward A, Osei-Bonsu K, Branchini C, et al Enhancing governance and health system accountability for people centered healthcare: an exploratory study of community scorecards in Afghanistan. BMC Health Serv Res. 2015;15:299. 26227814 10.1186/s12913-015-0946-5 PMC4521484

[12] Molyneux S, Atela M, Angwenyi V, et al Community accountability at peripheral health facilities: a review of the empirical literature and development of a conceptual framework. Health Policy Plan. 2012;27:541–554. 22279082 10.1093/heapol/czr083 PMC3465752

[13] Ringhold F, Holla A, Koziol M, et al Citizens and service delivery: assessing the use of social accountability approaches in human development sector. Washington, DC: World Bank; 2012.

[14] McCoy DC, Hall JA, Ridge M A systematic review of the literature for evidence on health facility committees in low- and middle-income countries. Health Policy Plan. 2012;27:449–466. 22155589 10.1093/heapol/czr077

[15] Joshi A Reading the local context: a causal chain approach to social accountability. IDS Bull. 2014;45:23–35.

[16] Joshi A Do they work? Assessing the impact of transparency and accountability initiatives in service delivery. Dev Policy Rev. 2013;31:s29-s48.

[17] Evans T, Chowdhury A, Evans D, et al Thailand’s universal coverage scheme: achievements and challenges. An independent assessment of the first 10 years (2001-2010). Nonthaburi, Thailand: Health Insurance System Research Office; 2012 120p.

[18] Tangcharoensathien V, Pitayarangsarit S, Patcharanarumol W, et al Promoting universal financial protection: how the Thai universal coverage scheme was designed to ensure equity. Health Res Policy Syst. 2013;11:25. 23919275 10.1186/1478-4505-11-25 PMC3735425

[19] Arredondo A, Orozco E, Aviles R Evidence on equity, governance and financing after health care reform in Mexico: lessons for Latin American countries. Saúde Sociedade. 2015;24:162–175.

[20] OECD OECD review of health systems: Mexico 2016. Paris: OECD Publishing; 2016.

[21] Dasgupta R, Nandi S, Kanungo K, et al What the good doctor said: A critical examination of design issues of the RSBY through provider perspectives in Chhattisgarh, India. Soc Change. 2013;43:227–243.

[22] Nandi S, Kanungo K, Khan MH, et al A study to analyse implementation of RSBY in Chhattisgarh. BMC Proc. 2012;6:05.

[23] Onwujekwe O, Obi F, Uzochukwu B Assessment of the NHIS-MDG free maternal and child health program and the prospects of reactivation/scale-up using the basic health care provision fund in Nigeria. Research Summary 7. Enugu, Nigeria: Health Policy Research Group, University of Nigeria; 2016 9.

[24] Garza B Increasing the responsiveness of health services in Mexico’s Seguro Popular: three policy proposals for voice and power. Health Syst Reform. 2015;1:235–245. 10.1080/23288604.2015.1059538 31519077

[25] McMahon SA, George AS, Chebet JJ, et al Experiences of and responses to disrespectful maternity care and abuse during childbirth; a qualitative study with women and men in Morogoro Region, Tanzania. BMC Pregnancy Childbirth. 2014;14:268. 25112432 10.1186/1471-2393-14-268 PMC4261577

[26] Atela M, Bakibinga P, Ettarh R, et al Strengthening health system governance using health facility service charters: a mixed methods assessment of community experiences and perceptions in a district in Kenya. BMC Health Serv Res. 2015;15:539. 26637186 10.1186/s12913-015-1204-6 PMC4670501

[27] Sato M, Gilson L Exploring health facilities’ experiences in implementing the free health-care policy (FHCP) in Nepal: how did organizational factors influence the implementation of the user-fee abolition policy? Health Policy Plan. 2015;30:1272–1288. 25639824 10.1093/heapol/czu136

[28] Belaid L, Ridde V An implementation evaluation of a policy aiming to improve financial access to maternal health care in Djibo district, Burkina Faso. BMC Pregnancy Childbirth. 2012;12:143. 23216874 10.1186/1471-2393-12-143 PMC3538061

[29] Opwora A, Kabare M, Molyneux S, et al Direct facility funding as a response to user fee reduction: implementation and perceived impact among Kenyan health centres and dispensaries. Health Policy Plan. 2010;25:406–418. 20211967 10.1093/heapol/czq009 PMC2929466

[30] Okonofua F, Lambo E, Okeibunor J, et al Advocacy for free maternal and child health care in Nigeria - results and outcomes. Health Policy. 2011;99:131–138. 20727612 10.1016/j.healthpol.2010.07.013

[31] Muoghalu CO Socio-economic and cultural factors in maternal mortality in Nigeria. Gend Behav. 2010;8:3226–3239.

[32] Akeju DO, Oladapo OT, Vilder M, et al Determinants of health care seeking behaviour during pregnancy in Ogun State, Nigeria. Reprod Health. 2016;13:32. 27356754 10.1186/s12978-016-0139-7 PMC4943510

[33] Sambo MN, Abdulrazak GA, Shamang AF, et al Household cost of antenatal care and delivery services in a rural community of Kaduna state, northwestern Nigeria. Niger Med J. 2013;54:87. 23798792 10.4103/0300-1652.110034 PMC3687870

[34] Adedini SA, Odimegwu C, Bamiwuye O, et al Barriers to accessing health care in Nigeria: implications for child survival. Glob Health Action. 2014;7:23499. 10.3402/gha.v7.23499 PMC3957799 24647128

[35] Aniebue N, Ugbene E PATHS2 consultancy report on revision of FMCH policy and implementation guidelines in Enugu state. Enugu, Nigeria: PATHS2; 2013.

[36] Okeibunor JC, Onyeneho NG, Okonofua FE Policy and programs for reducing maternal mortality in Enugu State, Nigeria. Afr J Reprod Health. 2010;14:19–30.

[37] State Ministry of Health [SMOH] [Enugu Nigera] The operational guidelines for health facility committees in Enugu State. Enugu, Nigeria: Enugu State Ministry of Health; 2010.

[38] Brinkerhoff DW, Bossert TJ Health governance: concepts, experience, and programming options. Bethesda, MD: Health Systems 20/20; 2008.

[39] Brinkerhoff DW, Bossert TJ Health governance: principal–agent linkages and health system strengthening. Health Policy Plan. 2014;29:685–693. 23411121 10.1093/heapol/czs132

[40] Abimbola S, Molemodile SK, Okonkwo OA, et al ‘The government cannot do it all alone’: realist analysis of the minutes of community health committee meetings in Nigeria. Health Policy Plan. 2015;31:332–345. 26210167 10.1093/heapol/czv066 PMC4779146

[41] Abimbola S, Ogunsina K, Charles-Okoli AN, et al Information, regulation and coordination: realist analysis of the efforts of community health committees to limit informal health care providers in Nigeria. Health Econ Rev. 2016;6:51. 27844451 10.1186/s13561-016-0131-5 PMC5108730

[42] Uzochukwu B, Onwujekwe O, Ezumah N The district health system in Enugu State: an analysis of policy development and implementation. Afr J Health Econ. 2014;3:1–14.

[43] Nigeria Population Commission Nigeria demographic and health survey (NDHS) 2013. Abuja, Nigeria: National Population Commission and ICF International; 2014.

[44] Gilson L Health policy and systems research: A methodological reader. Geneva, Switzerland: Alliance for Health Policy and Systems Research, World Health Organization; 2012.

[45] Yin RK Case study research: design and methods. 4th ed. Los Angeles, CA: Sage Publications; 2009.

[46] Yin R Qualitative research from start to finish. New York & London: The Guilford Press; 2011.

[47] Harper M, Cole P Member checking: can benefits be gained similar to group therapy? Qual Rep. 2012;17:510–517.

[48] QSR International Pty Ltd NVivo qualitative data analysis software. Version 11. Melbourne, Australia: QSR International Pty Ltd; 2015.

[49] Pritchard K, Whiting R Autopilot? A reflexive review of the piloting process in qualitative e-research. Qual Res Organ Manage Int J. 2012;7:338–353.

[50] Creswell JW Qualitative enquiry and research design: choosing among five approaches. 2nd ed. Thousand Oaks, CA: Sage Publications; 2007.

[51] Jongudomsuk P, Srithamrongsawat S, Patcharanarumol W, et al The Kingdom of Thailand health system review 2015. Manila, Philippines: World Health Organisation, Regional Office for the Western Pacific; 2016.

[52] Anwari Z, Shukla M, Maseed BA, et al Implementing people-centred health systems governance in 3 provinces and 11 districts of Afghanistan: a case study. Confl Health. 2015;9:2. 25904978 10.1186/1752-1505-9-2 PMC4406217

[53] Starr MA Qualitative and mixed‐methods research in economics: surprising growth, promising future. J Econ Surv. 2014;28:238–264.

